# Mandibular Arteriovenous Malformation with Unusual Radiographic Appearance

**DOI:** 10.30476/DENTJODS.2021.90822.1526

**Published:** 2023-03

**Authors:** Nafiseh Shamloo, Fatemeh Mashhadiabbas, Roohallah Safarpour

**Affiliations:** 1 Dept. of Oral and Maxillofacial Pathology, Dental School, Shahid Beheshti University of Medical Sciences, Tehran, Iran; 2 Postgraduate Student, Dept. of Oral and Maxillofacial Pathology, Dental School, Shahid Beheshti University of Medical Sciences, Tehran, Iran

**Keywords:** Arterio-venous malformation, Mandible, Treatment

## Abstract

Arteriovenous malformation of head and neck is a rare vascular anomaly but when present, it is persistent and progressive in nature. It can also represent a lethal benign disease due to massive hemorrhage. There are several indications for treatment including age, location, extension and type of vascular malformation. Endovascular therapy can effectively cure most lesions with limited tissue involvement. Surgery can be used in selected cases in combination with embolization. Here, we present a rare case of arteriovenous malformation of mandible with floating tooth appearance in an 11-year-old boy patient. Given the spectrum of imaging presentations seen and the crossover with other lesions, microscopic histopathological examination is the gold standard for diagnosis.

## Introduction

All vascular lesions until 1980's were referred to as hemangiomas. Classification of vascular lesions based on endothelial characteristics into hemangiomas and vascular malformation was done by Mullikin *et al*. [ 1 ] in 1982. In 1996, International Association of the Study of Vascular Abnormalities modified the classification as vascular tumors or vascular malformations (VMs) [ 2 ]. VMs based on blood flow are classified into lesions with low flow such as lymphatic, capillary, venous malformations also lesions with high-flow lesions such as arteriovenous malformations (AVMs) as well as arteriovenous fistulas [ 1 ]. Differentiation criteria between AVM and hemangioma are based on the clinical and histological characteristics. In general, there is no sign of hemangiomas at birth; they are characterized by a rapid growth phase with endothelial cell proliferation. Shortly after birth, hemangiomas grow fast usually faster than the child's growth, which then show a gradual involution. On the other hand, symptoms of AVMs are visible and they grow relative to the growth of the child and remain throughout the life. They are characterized by the normal turnover rate of endothelial cell and direct, permanent communication between arteries and veins [ 1 , 3 ]. As benign lesions, AVMs can arise in the any area of the body. In some rare cases, AVMs appear in hard tissue like bone/jaws and cause a highly unusual spectrum of symptoms [ 4 - 5 ]. Jaw intraosseous AVMs have a high risk in life threatening hemorrhage either spontaneously or following oral surgeries [ 6 - 7 ]. In this article, we report the case of mandibular intraosseous AVMs in an 11-year-old boy with floating tooth appearance in radiographic feature.

## Case Presentation

**Figure 1:**
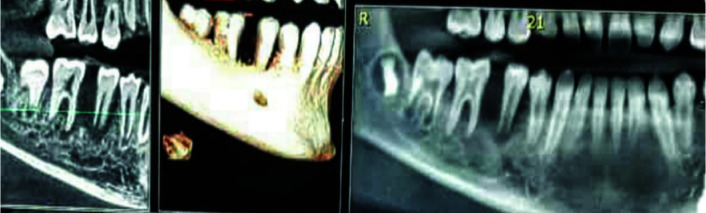
Floating tooth appearance

**Figure 2:**
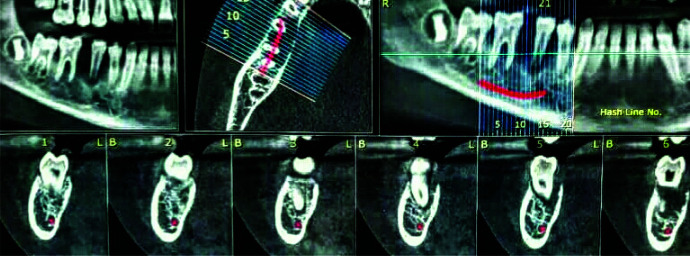
Cone beam computed tomography (CBCT) image; mandibular canal is intact

An 11-year-old boy with complaint of a swelling with out pain of mandibular right posterior area and tooth mobility in that region for one month was referred to the Department of Oral and Maxillofacial Surgery, Shahid Beheshti University of Medical Sciences, Tehran, Iran. The patient did not report a history of trauma or medical problems. Clinical examination was normal. In the intraoral examination, a fluctuant swelling extending from first premolar to first molar with erythematous mucosa was seen. Cone beam computed tomography (CBCT) showed an ill-defined unilocular radiolucency that extended from first molar to the distal of first premolar with floating tooth appearance (Figure 1). Bone destruction was seen in lingual and buccal crest, which caused perforation in some areas. It seemed that mandibular canal was intact (Figure 2). On aspiration, blood was detected. Due to clinical, radiographic and aspiration findings, central giant cell granuloma, leukemia, and osteosarcoma were suggested as a differential diagnosis. Under local anesthesia, excisional biopsy of the lesion was done; it was completely excised with two teeth and submitted for the histologic examination. The gross appearance was an irregular, creamy-brown soft tissue with elastic consistency, measuring 2.5×2×1 centimeters.

**Figure 3:**
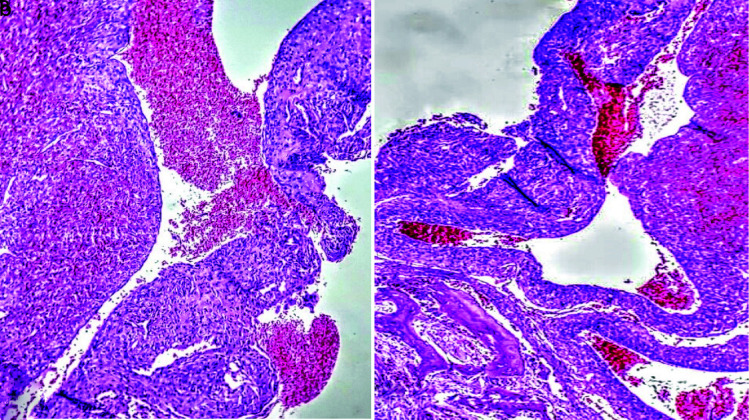
along with capillary vessels (200 ×Magnification, H&E)

**Figure 4:**
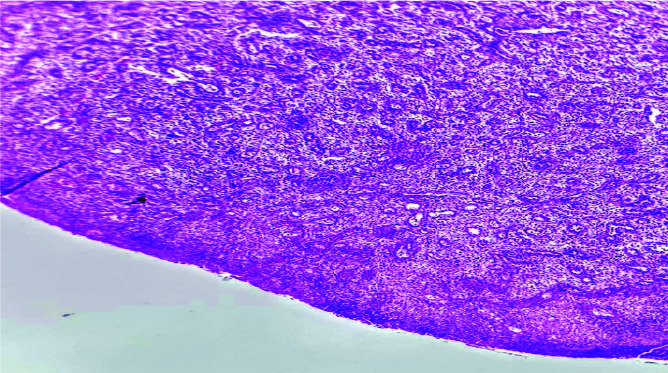
Photomicrograph showing an ulcerated surface epithelium (100 ×Magnification, H&E)

**Figure 5:**
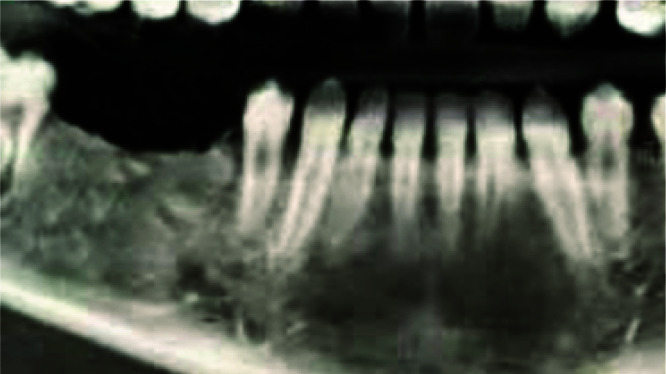
Dental panoramic radiograph of the patient at follow up revealed obviously bone repair of alveolus

Histopathological sections showed a vascular lesion composed of a varying mixture of arteries, veins, and associated small vessels within a fibrous background. Mixed inflammatory cells infiltration, giant cells, hemorrhage, and curetted bone are seen (Figure 3a and b). The lesion was covered by keratinized epithelium, which in some areas was ulcerated and replaced by fibrinopurulent membrane (Figure 4). According to histopathologic and radiographic findings, the diagnosis of AVM was done. There was no recurrence during a 6-month follow-up (Figure 5).

## Discussion

The VMs are malformed communications which lined by inactive endothelium. While such conditions are generally congenital, they demonstrate an extra growth if disturbed by infections, trauma or endocrine fluctuations [ 8 ]. Deepa *et al*. [ 9 ] indicated that the average age of presentation of intraosseous arteriovenous malformations in jaws (j-AVMs) is 19 years. In the current case, there were no symptoms until 11 years when tooth mobility was noticed. J-AVMs are very rare conditions but featured a higher tendency of life-threatening due to hemorrhage either spontaneously or due to surgery, therefore differential diagnosis must be taken into account in deciding about unexplained symptoms and pathology [ 4 , 10 ]. The prevalence of the complication in female is twice that of male, while in our case is male, with the highest incidence between the ages of 10 and 20 with extremes at 3 months and 74 years of age [ 11 ]. The head and neck region constitutes 50% of vascular lesions so that only a small number of cases happen in the jaws. In addition, the prevalence of the complication is twice in the mandible than maxilla, as we observed in this case [ 11 ]. AVMs have slow growth nature, for this reason, they are asymptomatic for long time and can be seen at any age [ 12 ]. They usually appear without symptoms like bruit, swelling of soft tissues, dental loosening, altered color of skin and mucosa and paresthesia of the lower lip or chin [ 13 , 14 ]. Although this case has no history of dental visits, but the appearance of jaw swelling and tooth mobility has been observed suddenly during one month. Due to existence of teeth, complex morphology of jaw structures and vital structures in maxillofacial area, diagnosis and management of jaw AVMs are more difficult than other intrabony AVMs [ 6 ]. When AVM appears close to teeth, they create mobility and displacement, consistent with the slow-growing nature, like in the present case. Cases of root resorption have also been reported [ 15 ]. In our case, bone destruction is seen in buccal and lingual crest, noticeably erosion of the alveolar process with apparently floating teeth, but root resorption was not detected. Clinically, J-AVMs are seen following extractions, while brushing or biopsy procedures [ 8 ]. The radiographic appearances of these lesions are variable and can be similar to other lesions; therefore, having a definitive diagnosis based just on imaging is not easy [ 16 ]. Differential diagnosis of J-AVMs includes hemangioma, Langerhans’s cell histiocytosis, simple bone cyst, Ewing’s sarcoma, aneurysmal bone cyst, ameloblastoma, osteosarcoma, odontogenic myxoma, ameloblastic fibroma, central giant cell granuloma, odontogenic keratocyst, dentigerous, fibrous dysplasia and metastatic malignant tumors [ 8 , 17 - 18 ]. Despite of unilocular radiolucent lesion in our case, most commonly, these lesions show a multilocular radiolucent defect. The lesions can be large (soap bubble appearance) or small (honeycomb appearance) [ 19 ]. In addition, the lesions may appear as an ill-defined radiolucent area or a well-defined, cystic-radiolucency. In the case of larger malformation, cortical expansion or a sunburst radiographic appearance is expected. It is possible to demonstrate the vascular nature of the lesions using angiography [ 19 ].

In few cases, there are notable osseous erosions of the alveolus with visible floating teeth [ 20 ], as such in the present case. These features can be seen in other lesions like ameloblastomas, osteosarcomas, myxomas, and fibrous dysplasia [ 21 ]. Biopsy and histopathological examination for precise diagnosis of AVMs is needed. Features of AVM histology are relatively similar, so that fibrous tissue and proliferative epithelial cells are usually found around the lesion and the remaining bony trabeculae are interspersed with endothelial cells and vascular structures [4,22], as the present case which the remaining bony trabeculae were interspersed with vascular structures (Figure 3b).

AVMs demonstrate a therapeutic challenge because of their hemodynamic characteristics and their modality of growth. Elimination of lesion, hemorrhage control during surgery and preventing of recurrence are main goals for treatment of AVMs. Radiotherapy may be only treatment option for inaccessible AVMs; however, any deformity of the bone can be corrected using surgical approaches [ 23 ]. The sort of treatment also depends on size of lesion and degree of involvement of vital structures. Direct surgery is the standard treatment, which might be accompanied by embolization of major afferent (feeding) vessels. For lesions that require resection, radiographic embolization often is performed one to two days earlier than surgery process to minimize blood loss [ 19 ]. The present case, depending on the size of lesion, was also treated with direct surgery without embolization.

Through radical excision of the large lesion, we can perform curative purpose and in general, reconstruction is carried out immediately using a tissue graft [ 24 ].

The patient has signed informed consent for the surgical procedure and necessary information for reporting this case.

## Conclusion

Here, we presented a rare case of mandibular intraosseous AVM with floating tooth appearance in an 11-year-old male patient. Taking into account the spectrum of imaging presentations observed and the crossover with other lesions, microscopic histopathological investigation is the gold standard for precise diagnosis.

## Conflict of Interest

The authors declare that they have no conflict of interest.
